# Heat-Induced Calcium Leakage Causes Mitochondrial Damage in *Caenorhabditis elegans* Body-Wall Muscles

**DOI:** 10.1534/genetics.117.202747

**Published:** 2017-05-31

**Authors:** Kenta Momma, Takashi Homma, Ruri Isaka, Surabhi Sudevan, Atsushi Higashitani

**Affiliations:** Graduate School of Life Sciences, Tohoku University, Sendai 980-8577, Japan

**Keywords:** calcium, DAF-16, heat stress, mitochondrial fragmentation, ryanodine receptor

## Abstract

Acute onset of organ failure in heatstroke is triggered by rhabdomyolysis of skeletal muscle. Here, we showed that elevated temperature increases free cytosolic Ca^2+^ [Ca^2+^]f from RYR (ryanodine receptor)/UNC-68 *in vivo* in the muscles of an experimental model animal, the nematode *Caenorhabditis elegans*. This subsequently leads to mitochondrial fragmentation and dysfunction, and breakdown of myofilaments similar to rhabdomyolysis. In addition, treatment with an inhibitor of RYR (dantrolene) or activation of FoxO (Forkhead box O)/DAF-16 is effective against heat-induced muscle damage. Acute onset of organ failure in heatstroke is triggered by rhabdomyolysis of skeletal muscle. To gain insight into heat-induced muscle breakdown, we investigated alterations of Ca^2+^ homeostasis and mitochondrial morphology *in vivo* in body-wall muscles of *C. elegans* exposed to elevated temperature. Heat stress for 3 hr at 35° increased the concentration of [Ca^2+^]f, and led to mitochondrial fragmentation and subsequent dysfunction in the muscle cells. A similar mitochondrial fragmentation phenotype is induced in the absence of heat stress by treatment with a calcium ionophore, ionomycin. Mutation of the *unc-68* gene, which encodes the ryanodine receptor that is linked to Ca^2+^ release from the sarcoplasmic reticulum, could suppress the mitochondrial dysfunction, muscle degeneration, and reduced mobility and life span induced by heat stress. In addition, in a *daf-2* mutant, in which the DAF-16/FoxO transcription factor is activated, resistance to calcium overload, mitochondrial fragmentation, and dysfunction was observed. These findings reveal that heat-induced Ca^2+^ accumulation causes mitochondrial damage and consequently induces muscle breakdown.

HEATSTROKE is a life-threatening condition, the risk of which is increasing with a warming climate, the urban heat island effect, and an aging population (Patz *et al.* 2005; Luber and McGeehin 2008; Rowlands *et al.* 2012). Under mild heat stress resulting in an acute heat response, symptoms of dizziness, headache, and muscle cramps appear, then gradually subside upon shift-down to the permissive temperature. In severe cases leading to heatstroke, breakdown of muscle fiber (*i.e.*, rhabdomyolysis) occurs, which in turn causes acute renal failure through accumulation of myoglobin and finally death with multiple organ dysfunction (Abderrezak *et al.* 2002). Malignant hyperthermia is a life-threatening pharmacogenetic syndrome of hypermetabolism of skeletal muscle. It is primarily triggered in susceptible individuals by anesthetic agents and muscle relaxants, and is an inherited disorder caused by gain-of-function mutations in the skeletal muscle Ca^2+^ release channel ryanodine receptor (RYR). Persons susceptible to malignant hyperthermia are much more sensitive to external or environmental heatstroke and die a sudden death (Jurkat-Rott *et al.* 2000; Treves *et al.* 2005).

It is important to study the effects of heat on muscle cells to devise counteractive measures against heatstroke. Skeletal muscle contraction and relaxation are regulated by the concentration of free cytosolic Ca^2+^ ([Ca^2+^]f). Ca^2+^ is released from the sarcoplasmic reticulum (SR) store via RYR, and reuptake of Ca^2+^ into the SR is mediated via Sarco-Endoplasmic Reticulum Calcium ATPase (SERCA) (MacLennan 1990). The Ca^2+^ concentration in the SR and the endoplasmic reticulum (ER) is thousands of times greater than that in the cytosol (Berridge *et al.* 2003; Hajnóczky *et al.* 2003). Thermal instability of this Ca^2+^-sequestering property of the SR occurs via protein unfolding of muscle SERCA (Davidson and Berman 1996; Senisterra *et al.* 1997; Schertzer *et al.* 2002). High temperature also commonly leads to ER stress and activation of the unfolded protein response (UPR) (Schröder and Kaufman 2005; Ron and Walter 2007; Walter and Ron 2011).

The nematode *Caenorhabditis elegans* is a convenient model system for studying physiological processes at the molecular or genetic level. In addition, its body-wall muscle is analogous to vertebrate skeletal muscle in structure and function (Moerman and Fire 1997; Gieseler *et al.* 2017). *C. elegans* body-wall muscle generates all-or-none action potentials (Liu *et al.* 2011) with certain molecules including RYR (UNC-68 in *C. elegans*) and SERCA (SCA-1 in *C. elegans*), being highly conserved from the nematode to mammals (Maryon *et al.* 1996, 1998; Cho *et al.* 2000; Zwaal *et al.* 2001). Like mammalian muscle twitching due to heatstroke, *C. elegans* are paralyzed by heat stress (Furuhashi and Sakamoto 2014). In addition, high temperature causes alternative splicing of the *xbp-1* gene in ER stress with the UPR in *C. elegans*, similar to mammalian cells. The X-box binding protein 1 (XBP-1) transcription factor upregulates ER stress-protective genes (Henis-Korenblit *et al.* 2010), while the DAF-16/FoxO (Forkhead box O) transcription factor is involved in longevity and resistance to various stresses such as heat stress and ER stress (Kenyon *et al.* 1993; Lithgow *et al.* 1995; Liang *et al.* 2006; Henis-Korenblit *et al.* 2010). However, both in mammalian and nematode systems, it is still unclear if, upon heat stress, DAF-16 is involved in counteractive measures against heatstroke and general Ca^2+^ homeostasis in muscle cells.

Here, we show for the first time how heat stress results in mitochondrial fragmentation and muscle defects caused by an upregulation of the [Ca^2+^]f in *C. elegans*. We further show that the heat-induced Ca^2+^ overload was completely suppressed by blocking RYR/UNC-68 receptor function. Moreover, we observed suppression of heat-induced Ca^2+^ accumulation and mitochondrial fragmentation in *C. elegans* with constitutively activated DAF-16.

## Materials and Methods

### Strains and culture conditions

We followed standard procedures for *C. elegans* strain maintenance (Sulston and Brenner 1974). The following strains were used in this study: N2 wild-type, CB1370: *daf-2*(*e1370*), CF1038: *daf-16*(*mu86*), HI1002: *daf-16*(*mu86*) *ccIs4251*[*Pmyo-3*::*nucGFP*::*LacZ + Pmyo-3*::mitochondrial *GFP*] *muIs84*[*Psod-3*::*GFP*], HI1003: *daf-2*(*e1370*) *ccIs4251*[*Pmyo-3*::*nucGFP*::*LacZ + Pmyo-3*::mitochondrial *GFP*] *muIs84*[*Psod-3*::*GFP*], SD1347: *ccIs4251*[*Pmyo-3*::*nucGFP*::*LacZ + Pmyo-3*::mitochondrial *GFP*], RW1596: *myo-3*(*st386*) *stEx30*[*Pmyo-3*::*GFP*::*MYO3 + rol-6*(*su1006*)], HBR4: *goeIs3*[*Pmyo-3*::*GCaMP3.35*::*unc-54-3′utr*, *unc-119*], TR2171: *unc-68*(*r1162*), HI1004: *daf-2*(*e1370*) *goeIs3*[*Pmyo-3*::*GCaMP3.35*::*unc-54-3′utr*, *unc-119*], HI1005: *daf-16*(*mu86*) *goeIs3*[*Pmyo-3*::*GCaMP3.35*::*unc-54-3′utr*, *unc-119*], HI1006: *unc-68*(*r1162*) *ccIs4251*[*Pmyo-3*::*nucGFP*::*LacZ + Pmyo-3*::mitochondrial *GFP*], and HI1007: *unc-68*(*r1162*) *goeIs3*[*Pmyo-3*::*GCaMP3.35*::*unc-54-3′utr*, *unc-119*]. All nematode experiments were performed at 20° unless otherwise noted.

**Figure 1:**
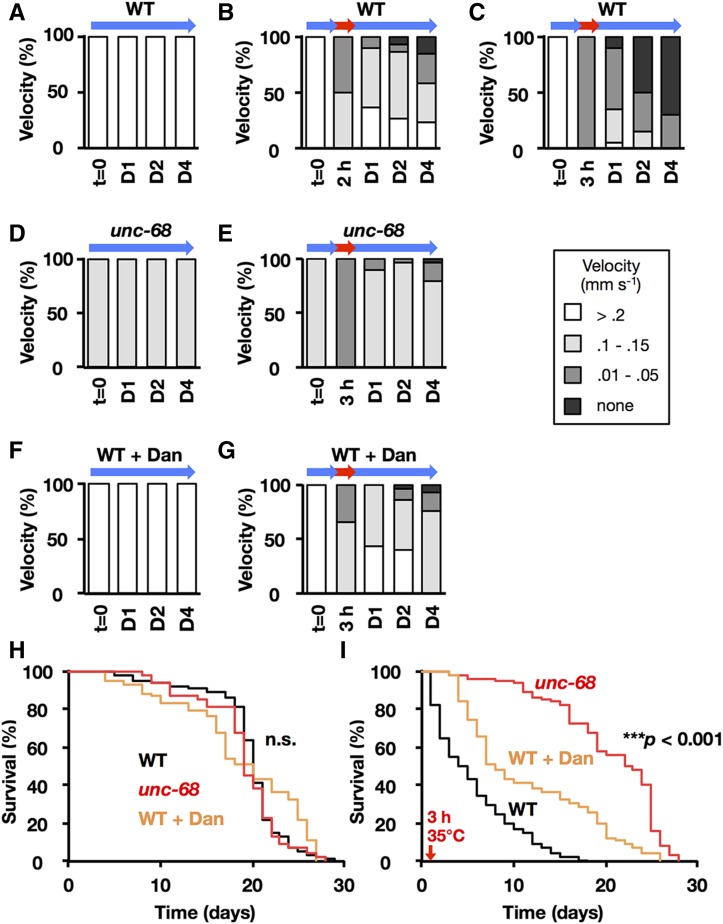
Velocities and survival rates after heat stress. (A–C) Velocity was measured in wild-type (WT) young-adult hermaphrodites that were cultured at 20° (A); or subjected to 2 hr of 35° (B) or 3 hr of 35° (C) and then shifted back down to 20° for 1 day (D1), 2 days (D2), or 3 days (D3). *t* = 0, control (time 0, 20°). (D and E) Velocity was measured in *unc-68* (*r1162*) young-adult hermaphrodites that were cultured at 20° (D), subjected to 3 hr of 35°, and then shifted back down to 20° (E). (F and G) Velocity was measured in WT worms treated with 100 µM Dantrolene (Dan), an inhibitor of RYR, at 20° (F), and after the worms were subjected to 3 hr of 35° and then shifted back down to 20° (G). (A–G) Shading represents values for velocity (mm sec^−1^): white, > 0.2; light gray, 0.1–0.15; gray, 0.01–0.05; and black, 0. *n* = 30 worms per condition. (H) Kaplan–Meier survival curves of WT (*n* = 113), *unc-68*(*r1162*) (*n* = 47), and WT treated with 100 µM Dan (*n* = 110) worms at 20°. (I) Kaplan–Meier survival curves of WT (*n* = 193), *unc-68*(*r1162*) (*n* = 101), and WT with 100 µM Dan (*n* = 95) worms subjected to 35° for 3 hr and 20° thereafter (*** *P* < 0.001 in all cases, log-rank test).

To study the effects of heat stress, synchronized young-adult hermaphrodites of wild-type N2, other mutants, and transgenic lines were transferred to NGM plates prewarmed at high temperature (35°) for 2 or 3 hr, by picking with a platinum wire pick. For synchronization, adult worms were placed on a NGM plate and removed after 6–8 hr. The eggs were monitored for 3 days and the young-adult worms were used for the experiment. After the heat stress, the velocity of the worms was measured, and then they were picked and transferred to fresh NGM plates at 20° for temperature shift-down (TSD) experiment. Velocity was calculated as the distance moved in 10 sec by using stereomicroscopy (SMZ18; Nikon, Garden City, NY) and imaging software (cellSensDP73; Olympus) after gently touching the worms (*n* = 30) with a platinum wire. A single group of animals was followed for 4 days after TSD. The velocity was classified into four categories: normal, > 0.2 mm/sec; slow, 0.1–0.15 mm/sec; severe (0.01–0.05 mm/sec; almost paralysis and only head moving); and dead (0 mm/sec; no response). The survival ratio after heat stress was also measured after TSD to 20° for > 40 worms as indicated in Figure 1. The worms were transferred to fresh NGM plates by picking them with a platinum wire pick every 2 days and observed until dead. The Kaplan–Meier survival curves were produced and long-rank statistical analysis was conducted by using BellCurve in Microsoft Excel. A single group of animals was followed for survival assay.

For treatment with the RYR/UNC-68 inhibitor dantrolene, synchronized L1 larvae were continuously cultured on a NGM plate with a final concentration of 100 µM dantrolene until the young-adult stage. Then, the young-adult worms were subjected to heat stress at 35° for 3 hr on a fresh NGM plate without dantrolene and monitored for survival rate, [Ca^2+^]f, and mitochondrial morphology in the body-wall muscle cells.

Ionomycin treatments (final concentrations 1 and 10 µM) were performed in S-basal solution with *Escherichia coli* OP50 (OD_600_ = 1.65) (Sulston and Brenner 1974). Three hours after incubation at 20°, the synchronized young-adult worms were washed twice with M9 buffer and then subjected to imaging analyses.

### Ca^2+^ imaging in body-wall muscle cells

[Ca^2+^]f in body-wall muscle cells was observed by assaying expression of the transgene *goeIs3*[*Pmyo-3*::*GCaMP3.35*::*unc-54-3′utr*, *unc-119*] (Schwarz *et al.* 2011). For each experiment, 10 worms were analyzed to obtain images of ∼50 muscle cells. GFP fluorescence was observed by using a BX51 fluorescent microscope (Olympus) with a DC73 CCD camera (Olympus) and an FV10i confocal laser-scanning microscope (Olympus). GFP intensity was measured using ImageJ software and the relative intensity was calculated by normalizing the average intensity with the intensity at *t* = 0.

### Imaging mitochondrial morphology and myofilaments in body-wall muscle cells

Mitochondrial morphology was observed with the transgene *ccIs4251*[*Pmyo-3*::*nucGFP*::*LacZ + Pmyo-3*::mitochondrial *GFP*]. The morphological categories were defined according to Regmi *et al.* (2014) with slight modification: (1) images indicating a majority of long interconnected mitochondrial networks were classified as *tubular*; (2) images indicating a combination of interconnected mitochondrial networks along with some smaller fragmented mitochondria were classified as *intermediate*; (3) images indicating a majority of short mitochondria were classified as *fragmented*; (4) images indicating sparse round mitochondria were classified as *swelling*; and (5) images indicating a collapse of muscular mitochondria and nuclei were classified as *cell death*. Body-wall muscle mitochondria were observed by using a confocal laser-scanning microscope (LSM 800; Zeiss [Carl Zeiss], Thornwood, NY).

To visualize F-actin in muscle cells, worms were washed off NGM plates with M9 buffer, collected in a 1.5 ml tube, and rinsed twice with M9 buffer. The worms were fixed in 1% paraformaldehyde for 10 min at room temperature, washed twice with PBS (pH 7.3), permeabilized with 100% cold acetone at −20° for 2 min, and finally rinsed twice with M9 buffer (Waterston *et al.* 1984). The worms were incubated for 2 hr at room temperature with a final concentration of 12 units/ml rhodamine phalloidin in ethanol (Molecular Probes, Eugene, OR). The myofibrils were observed by using a LSM 800 (Zeiss).

### Measurement of endogenous ATP

Six synchronized young-adult worms were collected in 100 µl of buffer (100 mM Tris-HCl, pH 7.5 and 40 mM EDTA), frozen in liquid nitrogen, boiled for 15 min, and homogenized with 0.3 g of ø 0.5 mm zirconia beads at 5000 rpm for 60 sec twice using a Micro Smash MS-100R homogenizer (Tomy Seiko, Japan). The supernatant was collected by centrifugation at 15,000 × *g* for 10 min, and ATP concentration was measured by using an ATP Determination Kit (Molecular Probes) and a multimode microplate reader (Spark 10 M; Tecan). Each experiment was performed in triplicate with three independent samples.

### ER stress monitored with xbp-1 splicing by quantitative RT-PCR

Total RNA was extracted from 300 young-adult worms with or without heat stress at 35° for 3 hr with TRI Reagent (Molecular Research Center), and treated with DNase (QIAGEN, Valencia, CA) to remove any contaminating genomic DNA. cDNA synthesis was performed using a PrimeScript RT reagent Kit (Takara). To detect both spliced and unspliced *xbp-1* transcripts, PCR was performed with Prime Star GXL (Takara) and a primer set surrounding the noncanonical intron of the *xbp-1* transcript: forward, 5′-TGC ATG CAT CTA CCA GAA CGT CGT CT-3′; and reverse, 5′-ATA GTT AGA TAC ATA TCC ACA CTG-3′ (Henis-Korenblit *et al.* 2010). The PCR products were separated by electrophoresis on a 3% MetaPhor gel (Lonza), stained with ethidium bromide, and quantified with ImageJ software. Real-time PCR experiments were performed in triplicate for each biological sample.

### Statistical analyses

Statistical analysis was performed in Microsoft Excel. Statistical significance was set at *P* < 0.05 using a Student’s two-tailed *t*-test.

### Data availability

Strains are available upon request. The authors state that all data necessary for confirming the conclusions presented in the article are represented fully within the article.

## Results

### Heat-induced motility reduction and recovery correlate with the [Ca^2+^]f level released through the ryanodine receptor UNC-68

The nematode *C. elegans* can grow and develop normally at 16–25° (Byerly *et al.* 1976). To study the effect of heat stress on motility, we analyzed the velocity of adult hermaphrodites exposed to heat shock at 35° for 2 or 3 hr. Based on the velocity, animals were classified into four categories: normal (> 0.2 mm/sec), slow (0.1–0.15 mm/sec), severe (0.01–0.05 mm/sec; head movement only), and dead (0 mm/sec; no response). Compared with the velocity at 20°, the velocity of wild-type N2 was lower after exposure to 35° for 2 hr (Figure 1, A and B); half of the population was almost paralyzed with severe movement defect, and the other half showed slow behavior. However, 24 hr after TSD to 20°, their movement was remarkably restored (Figure 1B). In contrast, the worms exposed to 35° for 3 hr did not recover, and ∼80% died within 4 days (Figure 1C).

**Figure 2:**
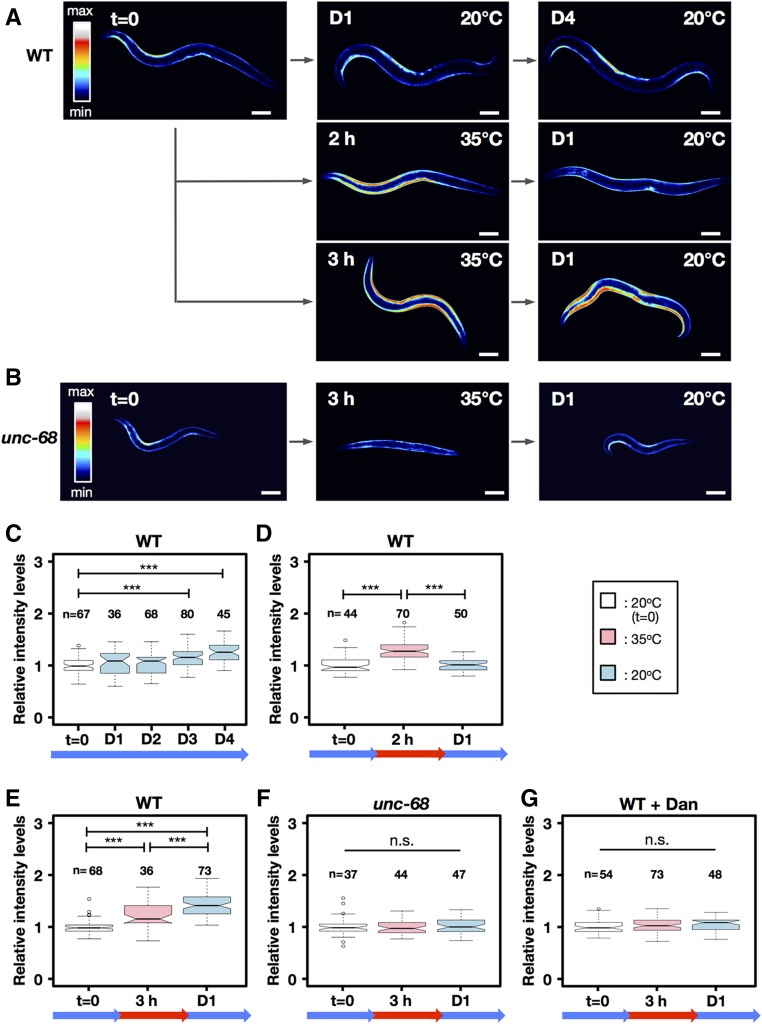
*In vivo* analysis of cytosolic calcium in transgenic animals expressing the *GCaMP3.35* Ca^2+^ indicator in body-wall muscle cells. Transgenic animals (*goeIs3*[*Pmyo-3*::*GCaMP3.35*::*unc-54-3′utr*, *unc-119*]) expressing GCaMP in body-wall muscle cells were monitored before and after heat treatment. (A) GCaMP-expressing young-adult hermaphrodites with a WT background were cultured at 20°, exposed to 2 or 3 hr of 35°, and shifted back down to 20°. The color of the bright signal in body-wall muscle cells indicates cytosolic free Ca^2+^ concentration. The images are representative of 10 independent experiments. Bar, 100 µm. *t* = 0 was taken as control. (B) GCaMP-expressing young-adult hermaphrodites with an *unc-68*(*r1162*) background were exposed to 35° for 3 hr and then shifted back down to 20°. The bright signal in body-wall muscle cells indicates high-Ca^2+^ concentration. The images are representative of 10 independent experiments. Bar, 100 µm. (C–G) Relative concentrations of body-wall muscle Ca^2+^ at 20° for 4 days in WT (C), after 35° for 2 hr treatment and 20° for 1 day in WT (D), after 35° for 3 hr and 20° for 1 day in WT (E) or *unc-68*(*r1162*) (F), and WT cultured with 100 µM dantrolene. *** *P* < 0.001 indicates a statically difference by a Mann–Whitney-Wilcoxon test. *n*, number of muscle images per condition. D, day; n.s., not significant; WT, wild-type.

We then monitored muscle [Ca^2+^]f in the *C. elegans* HBR4 strain, which expresses the calcium sensor GCaMP3.35 in striated body-wall muscles (Schwarz *et al.* 2011). The GCaMP3.35 signal intensity was typically highest at the contracting side of a body bend at 20° (Figure 2A: *t* = 0). The signal intensity gradually increased in an age-dependent manner at 20° (Figure 2C: D3 and D4). Intriguingly, when worms were exposed to transient heat stress at 35°, the intensity markedly increased in not only the contracting side but also the expanding side (Figure 2A). In worms subjected to heat stress for 2 hr, but not those subjected to heat stress for 3 hr, the elevated GCaMP3.35 intensities decreased to the normal level by 1 day after TSD to 20°, and motility could be recovered (Figure 2, A, D, and E). This suggests that Ca^2+^ homeostasis was disturbed by heat shock for 3 hr much more so than by heat shock for 2 hr.

The *unc-68* null mutants including *r1162* are viable with normal muscle ultrastructure, but have reduced normal body tension and locomotion (Maryon *et al.* 1996, 1998; Figure 1D). Furthermore, the muscle [Ca^2+^]f oscillation is slower than in wild-type N2, which leads to sluggish muscle contraction and relaxation, suggesting that UNC-68 is dispensable for excitation–contraction coupling but is essential for amplification of Ca^2+^ signals via Ca^2+^-induced Ca^2+^ release (Maryon *et al.* 1996, 1998). To clarify the effect of inhibiting Ca^2+^ release from RYR/UNC-68 on muscle damage, we analyzed velocity and [Ca^2+^]f after 35° heat treatment for 3 hr in the *unc-68* null mutant (*r1162*) HI1007. The motility of the mutant was transiently reduced after heat shock but recovered almost to pretreatment levels 1 day after TSD to 20° (Figure 1, D and E). In the *unc-68* (*r1162*) mutant, [Ca^2+^]f did not increase 1 day after TSD to 20° (Figure 2F). Moreover, the life span of the *unc-68* (*r1162*) mutant TR2171 was not shortened by 35° heat treatment for 3 hr, although that of wild-type N2 was drastically reduced (Figure 1, H and I). When wild-type N2 was cultured in the presence of 100 µM dantrolene, an inhibitor of RYR/UNC-68, the increase in [Ca^2+^]f induced by 35° heat treatment for 3 hr was suppressed (Figure 2, E and G), along with reductions in movement velocity and shortened life span (Figure 1, C and F–I).

### Heat-induced mitochondrial fragmentation caused by increased [Ca^2+^]f

**Figure 3:**
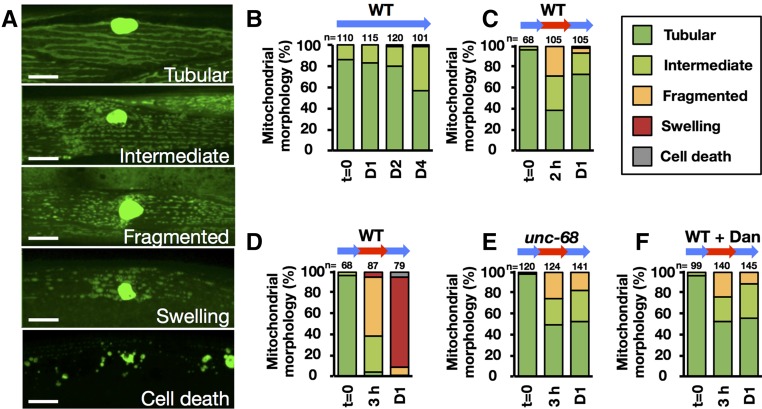
Heat-induced mitochondrial morphology alteration in body-wall muscle cells. Transgenic *C. elegans* (strain S1347) expressing mitochondria-targeted GFP and nuclear-targeted GFP–*LacZ* in body-wall muscle cells (*ccIs4251*[*Pmyo-3*::*GFP-LacZ* + *Pmyo-3*::mitochondrial *GFP*]) were monitored after heat stress. (A) Representative images of the mitochondrial morphologies observed. Bar, 10 µm. (B–F) Mitochondrial morphology was quantified at 20° for 1, 2, and 4 days (D1, D2, and D4) [(B): wild-type (WT)], 35° treatment for 2 hr and then 20° for 1 day [(C): WT], and 35° treatment for 3 hr and then 20° for 1 day [(D): WT, (E): *unc-68*(*r1162*), and (F): WT with 100 µM dantrolene (Dan)]. Green, light green, orange, red, and gray indicate percentage of worms with mitochondria classified as Tubular, Intermediate, Fragmented, Swelling, or Cell death. *n*, number of muscle mitochondria images per condition.

To investigate the causal relationship between the reduction of movement activity, increased [Ca^2+^]f, and a possible alteration of mitochondrial morphology in muscle cells exposed to heat, we examined mitochondrial and nuclear GFP signals in muscle cells of *C. elegans* strain SD1347 (Sarasija and Norman 2015). In the control worms, muscle mitochondria showed a tubular morphology (Figure 3A). In worms kept at 20° for 4 days, mitochondrial fragmentation slowly increased with the aging process (Figure 3B). In contrast, when temperature was elevated to 35°, mitochondrial fragmentation occurred rapidly (*i.e.*, within 2 hr; Figure 3C). After TSD to 20° for 1 day, the fragmentation was well recovered (*i.e.*, returned to > 70% tubular morphology) in the muscle cells of 2-hr heat-treated worms, but the damage was exacerbated to the point of mitochondrial swelling in the muscle cells of 3-hr heat-treated worms (Figure 3, C and D).

**Figure 4:**
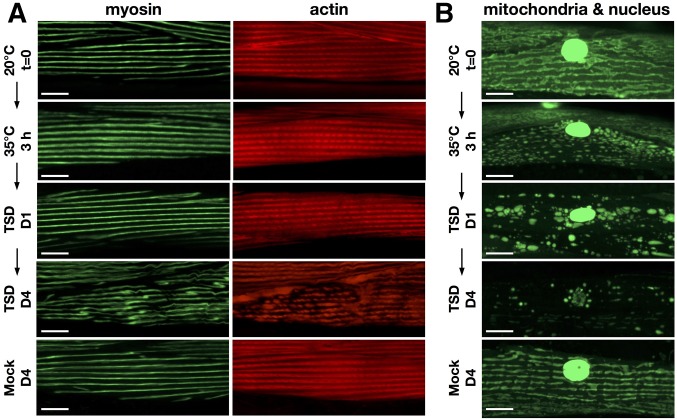
Visualization of muscle fiber and mitochondria. (A) Transgenic *C. elegans* (strain RW1596) expressing a GFP-tagged muscle Myosin (*stEx30*[*Pmyo-3*::*GFP*::*MYO3*]) were stained by rhodamine–phalloidin to visualize thick filaments and thin filaments in muscle cells, and observed after heat stress or without heat temperature stress (Mock). (B) Transgenic *C. elegans* (strain SD1347) expressing mitochondria-targeted GFP and nuclear-targeted GFP–*LacZ* in body-wall muscle cells (*ccIs4251*[*Pmyo-3*::*GFP-LacZ* + *Pmyo-3*::mitochondrial *GFP*]) were monitored after heat stress or without heat temperature stress (Mock). Bar, 10 µm. TSD, temperature shift-down.

Disorganization and degradation of muscle fiber (actin and myosin filaments) was not observed after 1 day of TSD to 20° following 3-hr heat treatment, although mitochondrial fragmentation and swelling was observed (Figure 4, TSD D1). Muscle damage was observed much later after 4 days of TSD (Figure 4A, TSD D4). We also observed decreased endogenous ATP levels in wild-type worms 1 day after TSD following 3-hr heat treatment, but not 2-hr treatment (Supplemental Material, File S1 and Figure S1).

**Figure 5:**
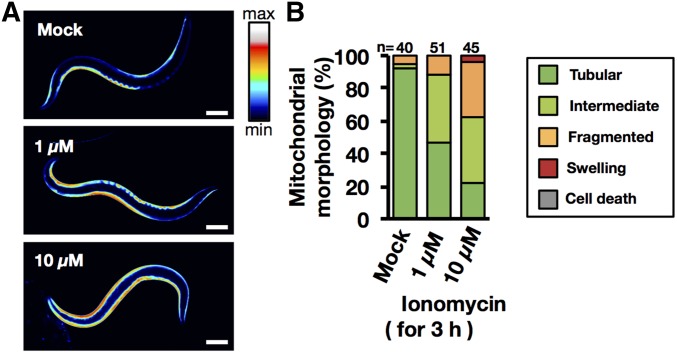
Influence of ionomycin on body-wall muscle mitochondria. Transgenic *C. elegans* (strain HBR4) expressing GCaMP in body-wall muscle cells (*goeIs3*[*Pmyo-3*::*GCaMP3.35*::*unc-54-3′utr*, *unc-119*]) were observed after ionomycin treatment. (A) Typical images of muscular Ca^2+^ concentration after vehicle control (DMSO) or ionomycin (1, 10 µM) treatment for 3 hr. The bright signal in the body-wall muscle cells indicates high-Ca^2+^ intensity. Bar, 100 µm. (B) Quantification of mitochondrial morphology was measured after vehicle control (DMSO) or ionomycin (1, 10 µM) treatment for 3 hr. Green, light green, orange, red, and gray indicate percentage of worms with mitochondria classified as Tubular, Intermediate, Fragmented, Swelling, or Cell death. *n*, number of muscle mitochondria images per condition.

In the null mutant of *unc-68* (*r1162*) HI1006 and the wild-type SD1347 treated with dantrolene, increase in [Ca^2+]^f as well as mitochondrial fragmentation was suppressed following 3-hr heat treatment 1 day after TSD (Figure 2, F and G and Figure 3, E and F). To determine whether increased [Ca^2+^]f could accelerate mitochondrial fragmentation in the *C. elegans* muscle cells, both HBR4 and SD1347 adult hermaphrodites were treated with the calcium ionophore ionomycin. After treatment with ionomycin for 3 hr, both GCaMP3.35 signal intensity and the progression of mitochondrial fragmentation increased in a dose-dependent manner (Figure 5).

Taken together, these results indicate that heat stress increases the concentration of muscle [Ca^2+^]f released from SR via RYR/UNC-68, which in turn accelerates muscle mitochondrial fragmentation and dysfunction in *C. elegans*.

### DAF-16/FoxO suppresses heat-induced Ca^2+^ leakage and mitochondrial fragmentation

**Figure 6:**
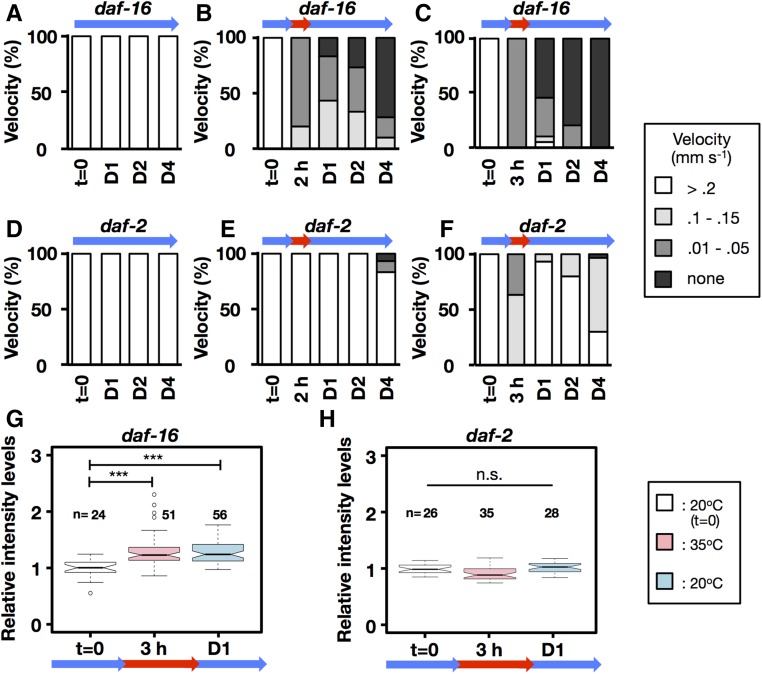
Thermotolerance and Ca^2+^ homeostasis brought about by DAF-16 activation. (A–F) Velocity was measured in *daf-16*(*mu86*) and *daf-2*(*e1370*) young-adult hermaphrodites that were cultured at 20° (A and D), shifted back down to 20° after 2 hr of 35° (B and E), or shifted back down to 20° after 3 hr of 35° (C and F). (G and H) Relative intensities of body-wall muscle Ca^2+^ signal after exposure to 35° for 3 hr and 20° for 1 day (D) [(G) *daf-16*(*mu86*) and (H) *daf-2*(*e1370*)]. Center lines show the medians; box limits indicate the 25th and 75th percentiles as determined by R software; whiskers extend 1.5 times the interquartile range from the 25th and 75th percentiles; outliers are represented by dots. *** *P* < 0.001 *vs.* control, Mann–Whitney–Wilcoxon test. *n*, number of muscle images per condition.

**Figure 7:**
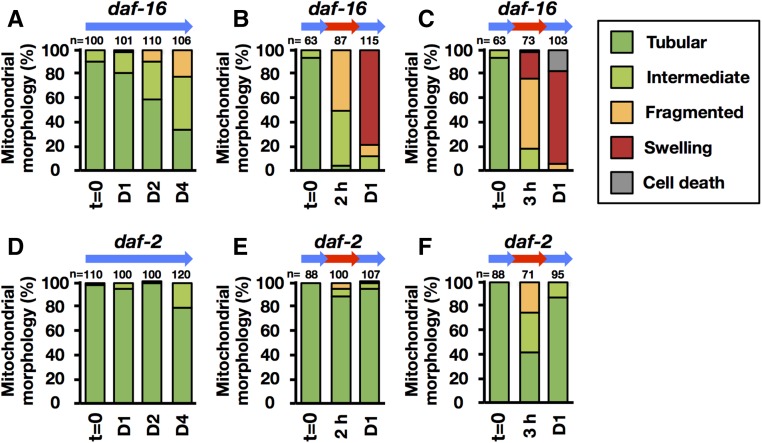
Retention of mitochondrial morphology and function brought about by DAF-16 activation. Transgenic *C. elegans* expressing *ccIs4251*[*Pmyo-3*::*GFP-LacZ* + *Pmyo-3*::mitochondrial *GFP*] in *daf-16*(*mu86*) (strain HI1002) and *daf-2*(*e1370*) (strain HI1003). (A–F) Mitochondrial morphology was quantified at 20° for 1, 2, and 4 days (D1, D2, and D4) (A and D), 35° treatment for 2 hr and 20° for 1 day (B and E), and 35° treatment for 3 hr and 20° for 1 day (C and F). Green, light green, orange, red, and gray indicate percentage of worms with mitochondria classified as Tubular, Intermediate, Fragmented, Swelling, or Cell death. *n*, number of muscle mitochondria images per condition.

DAF-16/FoxO is activated in the mutants of *daf-2* signaling, and it leads to tolerance to heat stress (Lithgow *et al.* 1995; Furuhashi and Sakamoto 2014). Here, we observed that *daf-16* mutant worms became hypersensitive and did not recover after heat treatment at 35° for 2 hr (Figure 6, A–C), unlike wild-type N2 under the same conditions (Figure 1B). In contrast, the *daf-2* mutant, which contains activated DAF-16/FoxO, could recover almost completely after heat treatment at 35° for 2 hr (Figure 7E). In addition, the *daf-2* mutant exposed to 35° for 3 hr could survive for almost 4 days (Figure 7D). Moreover, the heat-induced increase in [Ca^2+^]f and mitochondrial fragmentation, and decrease in endogenous ATP levels, observed in wild-type N2 (Figure 2, Figure 3, and Figure S1) were suppressed in the *daf-2* mutant and exacerbated in the *daf-16* mutant (Figure 6, Figure 7, and Figure S1).

**Figure 8:**
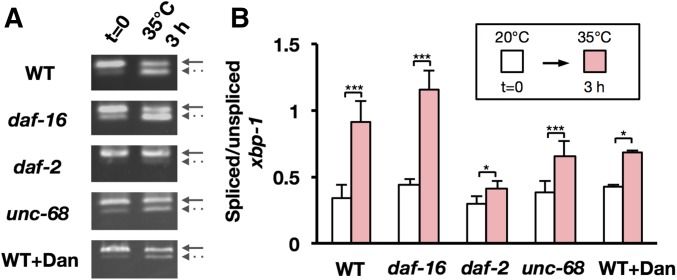
Alternative splicing of *xbp-1* mRNA in the unfolded protein response (UPR) (A) Representative RT-PCR products of unspliced *xbp-1* mRNA (solid arrows) and spliced *xbp-1* mRNA (dashed arrows). The *xbp-1* segment was amplified by a single set of PCR primers encompassing the putative intron region. (B) The bar graph shows the normalized mean ratio of spliced/unspliced *xbp-1* transcripts in three independent biological experiments. Data represent the mean of triplicate experiments ± SD (* *P* < 0.05, ** *P* < 0.01, *** *P* < 0.001, Student’s *t*-test). Dan, Dantrolene; WT, wild-type.

To study the relationship between the heat-induced UPR and an increase in [Ca^2+^]f, the level of the *xbp-1* splice variant, which is induced in response to the UPR (Shen *et al.* 2001; Calfon *et al.* 2002; Henis-Korenblit *et al.* 2010), was compared between wild-type N2 and the mutants *daf-2*, *daf-16*, and *unc-68*. In the wild-type N2, after heat stress at 35° for 3 hr, a shorter transcript of *xbp-1* mRNA was produced; the levels of this shorter transcript were much higher in the *daf-16* mutant and much lower in the *daf-2* mutant than in wild-type N2 (Figure 8). Intriguingly, the heat-induced *xbp-1* alternative splice variant was still observed in the *unc-68* mutant and in dantrolene-treated worms that suppressed heat-induced mitochondrial fragmentation (Figure 8). We also found that the expression levels of dynamin-related protein 1 (DRP-1) gene, which is involved in mitochondrial fission (Labrousse *et al.* 1999), were significantly induced in the wild-type N2 worms and the *daf-16* mutant, but not in the *daf-2* mutant 1 day after TSD following 3-hr heat treatment (Figure S2). These results suggest that increased [Ca^2+^]f is critical to accelerate mitochondrial fragmentation and dysfunction in *C. elegans* muscle cells, and that the DAF-16 and UPR pathways are important in protecting the cell against increased [Ca^2+^]f from heat shock.

## Discussion

In this study, we provide *in vivo* evidence that heat induces an increase in [Ca^2+^]f in muscle cells, which causes mitochondrial fragmentation leading to muscle cell dysfunction in *C. elegans*. The Ca^2+^ release from internal stores in the SR and ER is mediated by two channels, inositol (1,4,5) trisphosphate 3 kinase receptors [Ins(1,4,5)P3Rs] and RYRs, which are highly conserved in metazoans (Berridge *et al.* 2003). *C. elegans* has one ortholog of each of these: Ins(1,4,5)P3R/ITR-1 and RYR/UNC-68. The former is expressed in the intestine, pharynx, excretory cells, germline, spermatheca, and some neurons, and is required for pharynx pumping and the defecation cycle, while the latter is expressed in body-wall muscle cells and is required for normal body tension and locomotion (Maryon *et al.* 1998; Baylis *et al.* 1999). Here, we show that in worms exposed to 35°, [Ca^2+^]f immediately increased in the body-wall muscle cells. In contrast, the heat-induced increase in [Ca^2+^]f was significantly suppressed in the *unc-68* null mutant HI1007 and in wild-type HBR4 treated with the RYR/UNC-68 inhibitor dantrolene, clearly suggesting that RYR/UNC-68 has a major role in the Ca^2+^ release from the SR in body-wall muscle cells. At less than the threshold level of heat stimuli for 2 hr, Ca^2+^ levels and mitochondrial fragmentation were restored after TSD, and moving activity was concomitantly recovered. On the other hand, heat stress at 35° for 3 hr resulted in increase in the [Ca^2+^]f released from RYR/UNC-68 for a longer duration, and this calcium overload results in not only mitochondrial dysfunction but also muscle protein degradation. This consequently leads to reduced mobility and decreased life span. Although we could not explore the mechanism of calcium-mediated decrease in life span in this report, it may be possible that the Ca^2+^ overload activates a calcium-dependent protease calpain(s), which is involved in acute muscle protein degradation via dysfunction of integrin attachment complexes in *C. elegans* (Shephard *et al.* 2011; Etheridge *et al.* 2012). In mammalian skeletal muscle, calpains are thought to initiate cytoskeletal degradation by cleaving talin (Koh and Tidball 2000).

There are at least two possible mechanisms of heat-induced Ca^2+^ release from RYR/UNC-68: one direct and one indirect. Recent findings indicate that several amino acid substitutions in mammalian skeletal muscle RYR1 are associated with malignant hyperthermia, environmental heat stroke, and exercise-induced rhabdomyolysis (Wappler *et al.* 2001; Davis *et al.* 2002; Treves *et al.* 2005; Vladutiu *et al.* 2011). Therefore, we consider that, when exposed to temperature higher than the threshold temperature, RYR/UNC-68 with normal amino acid sequence might release Ca^2+^ directly. Another mechanism might involve heat inactivation of SERCA (SCA-1 in *C. elegans*), which is highly conserved between mammals and *C. elegans* [*e.g.*, 72% (721/999) identity and 82% (820/999) similarity between human SERCA1a and *C. elegans* SCA-1; 15 of the 24 cysteine resides of SERCA1a are conserved in SCA-1, indicated as a result of BLAST SEARCH on WormBase; Cho *et al.* 2000; Zwaal *et al.* 2001]. Elevated temperatures result in protein unfolding of mammalian SERCA, which exposes hydrophobic domains and leads to aggregation of Ca^2+^-ATPase monomers and the oxidation of sulfhydryl groups (Davidson and Berman 1996; Senisterra *et al.* 1997; Schertzer *et al.* 2002). These structural alterations result in an increase in the cellular Ca^2+^ level (Davidson and Berman 1996; Senisterra *et al.* 1997; Schertzer *et al.* 2002). Because we also observed UPR induction through *xbp-1* splicing in the wild-type worms exposed to 35° heat treatment, we propose that Ca^2+^ leakage may occur through SCA-1 and then activate additional Ca^2+^ release from RYR/UNC-68 in an indirect manner. RYRs function in Ca^2+^-induced Ca^2+^ release from SR for excitation–contraction coupling (Endo 1977; Zucchi and Ronca-Testoni 1997). We also found reduction of endogenous ATP levels 1 day after TSD following 3-hr heat treatment. This would further lead to an increase in the [Ca^2+^]f through inhibition of the Ca^2+^-ATPase SCA-1 activity.

The loss-of-function mutation of insulin-like receptor gene *daf-2* has been shown to increase longevity and resistance to various stresses, including heat stress (Kenyon *et al.* 1993; Lithgow *et al.* 1995; Liang *et al.* 2006; Furuhashi and Sakamoto 2014). Multiple transcription factors, such as FoxO/DAF-16 and HSF-1, are activated in the *daf-2* mutant (Hsu *et al.* 2003; Murphy *et al.* 2003). In addition, DAF-16 and XBP-1 collaborate to promote the ER stress response (Henis-Korenblit *et al.* 2010). As high temperature has been known to activate ER stress, we analyzed the increase in one of the ER stress markers, *xbp-1* splice variant (Henis-Korenblit *et al.* 2010). Here, we demonstrated that heat-induced *xbp-1* splice variant was lower in the *daf-2* mutant and much higher in the *daf-16* mutant when compared to wild-type N2. These results are in accordance with a previous report (Henis-Korenblit *et al.* 2010) that points toward the role of DAF-16 in protection against heat stress. In the case of the *daf-2* mutant, several protective mechanisms are already activated and hence heat stress does not lead to ER stress. On the other hand, the *daf-16* mutant is unable to activate any protective pathways, which in turn leads to an accumulation of unfolded proteins and consequently leads to ER stress. These results suggest that DAF-16 can inhibit the unfolding of RYR/UNC-68 and/or SERCA/SCA-1, leading to reduced [Ca^2+^]f, which suppresses the mitochondrial fragmentation and motility defect in the *daf-2* mutant. It may be directly related to longevity in the *daf-2* mutant.

Sarasija and Norman (2015) have reported that knockdown of *C. elegans* DRP-1, which is highly conserved in Metazoa (Ishihara *et al.* 2003; Cribbs and Strack 2007; Cereghetti *et al.* 2008; Han *et al.* 2008), suppresses mitochondrial fragmentation under [Ca^2+^]f overload conditions. Mammalian DRP-1 phosphorylation, dephosphorylation, and translocation to mitochondria are regulated by several Ca^2+^-dependent enzymes, such as Ca^2+^/calmodulin-dependent protein kinase, cyclin-dependent kinases, and calcineurin (Cereghetti *et al.* 2008; Han *et al.* 2008). In addition, alterations of the DRP-1 gene and protein expression are observed when mitochondrial fragmentation results from oxidative stress in mammals (Jendrach *et al.* 2008; Makino *et al.* 2010; Xu *et al.* 2013). DRP-1 upregulation is also observed in soleus muscle atrophy in hindlimb-unloaded mice (Cannavino *et al.* 2014). In our results, we found that expression of the *C. elegans drp-1* gene increased in the worms with compromised mitochondrial function: the expression levels were upregulated in the wild type and the *daf-16* mutant, but not in the *daf-2* mutant 1 day after TSD following 3-hr heat treatment (Figure S2). This result suggests that *drp-1* upregulation might exacerbate the mitochondrial damage in *C. elegans*.

In conclusion, we showed that elevated temperature increases [Ca^2+^]f from RYR/UNC-68 *in vivo* in the muscles of an experimental model animal, the nematode *C. elegans*. This subsequently leads to mitochondrial fragmentation and dysfunction, and breakdown of myofilaments similar to rhabdomyolysis. In addition, treatment with the RYR inhibitor dantrolene or activation of FoxO/DAF-16 is effective against heat-induced muscle damage.

## Supplementary Material

Supplemental material is available online at www.genetics.org/lookup/suppl/doi:10.1534/genetics.117.202747/-/DC1.
