# Supplementary material for: Heat-Induced Calcium Leakage Causes Mitochondrial Damage in Caenorhabditis elegans Body-Wall Muscles
Source: Genetics. 2017 May 31;206(4):1985–94. doi: 10.1534/genetics.117.202747 (PMC5560802; doi:10.1534/genetics.117.202747)
Supplement: Supplementary file 1 [file 1985FileS1.pdf]

Supplemental Figures

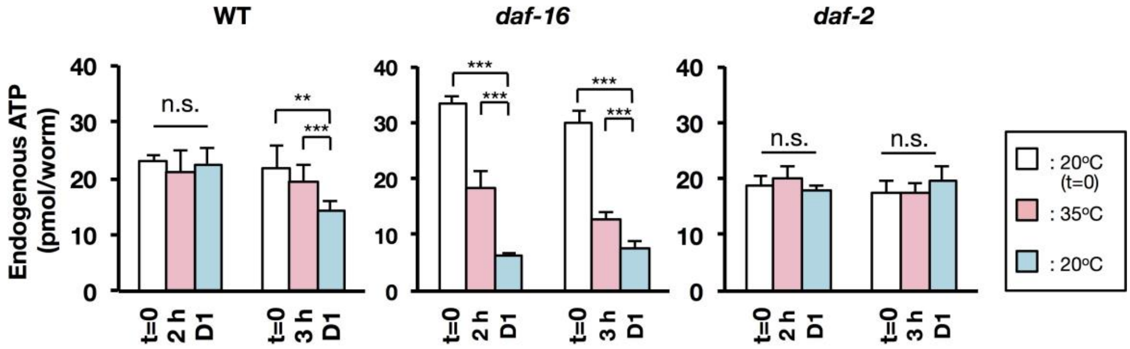

Supplemental Fig. S1. Alteration of endogenous ATP levels after heat stress. Endogenous ATP levels in wild-type N2, *daf-16*(*mu86*) and *daf-2*(*e1370*). ATP levels in whole body of worms (t=0: control at 20°C, 35°C for 2 or 3 h treatment, and 1 day after TSD to 20°C). Data are expressed as unit of *p*mol per worm. Six worms of each condition were collected for ATP measurement. Results represent mean of triplicate experiments ± SD ($^{**}P < 0.01$ and $^{***}P < 0.001$, Student's *t*-test).

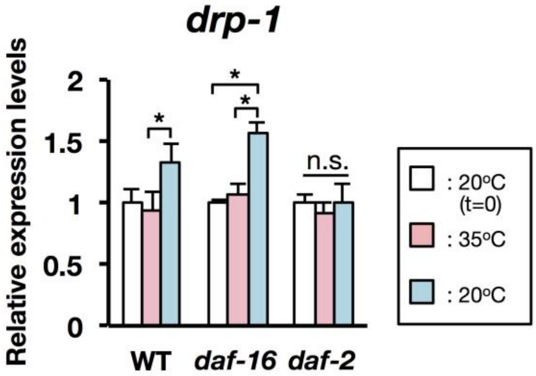

Supplemental Fig. S2. Real-time RT-PCR analysis of dynamin-related protein 1 (*drp-1*). The expression levels were monitored by real-time RT PCR using RNA samples from *C. elegans*. Adult synchronized wild-type N2, *daf-16*(*mu86*), and *daf-2*(*e1370*) worms were collected after 3 h of heat stress at 35°C and then 1 day after TSD to 20°C. Mean $\Delta\Delta C_T$ values were used to calculate relative changes in *drp-1* (5-ATG CAT CAA CCG GAG AAA GA-3, Rv: 5-TTG AAC GCT GTT CTT CAA CAA-3) expression normalized to levels of *act-1* (5-CAT GGC GGT ATG GGA CAG AA-3, Rv: 5-TCA ATT GGG TAC TTG AGG GTA-3). Results represent mean of relative *drp-1*/*act-1* ratios for triplicate experiments ± SD ($^{*}P < 0.05$, Student's *t*-test).
