# Supplementary figures and images for: Heat-Induced Calcium Leakage Causes Mitochondrial Damage in Caenorhabditis elegans Body-Wall Muscles
Source: Genetics. 2017 May 31;206(4):1985–94. doi: 10.1534/genetics.117.202747 (PMC5560802; doi:10.1534/genetics.117.202747)

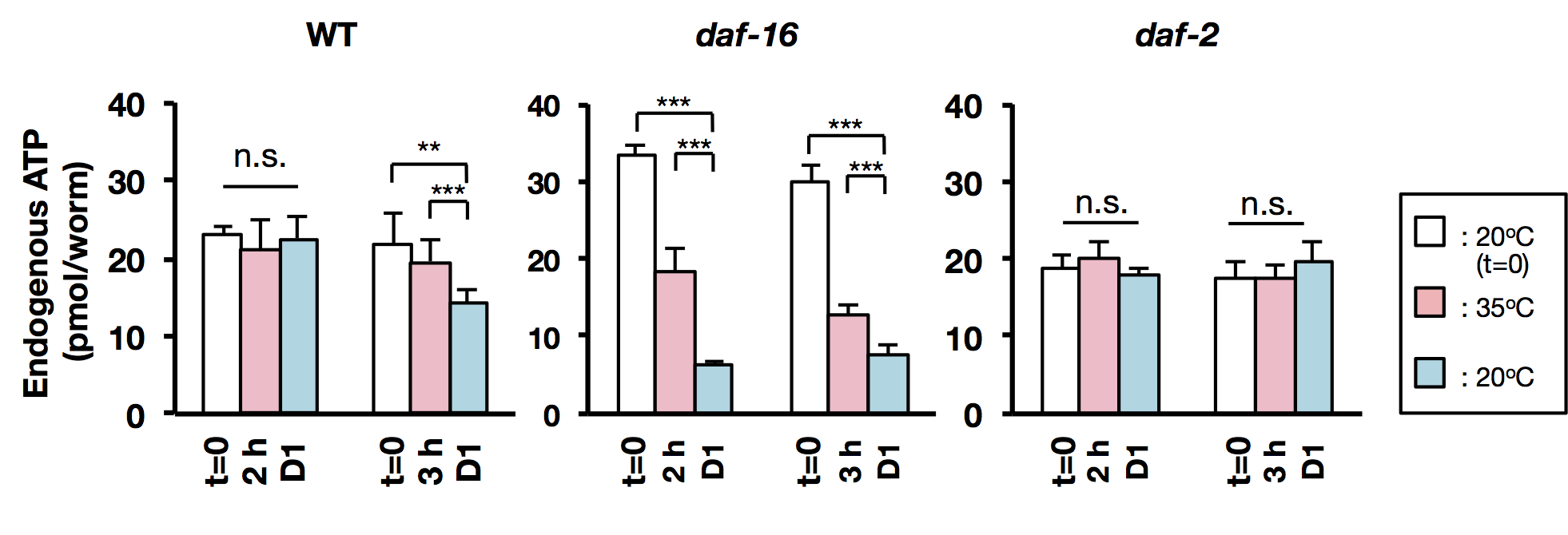

Supplement: Supplementary file 2 [file 1985FigureS1.tiff]

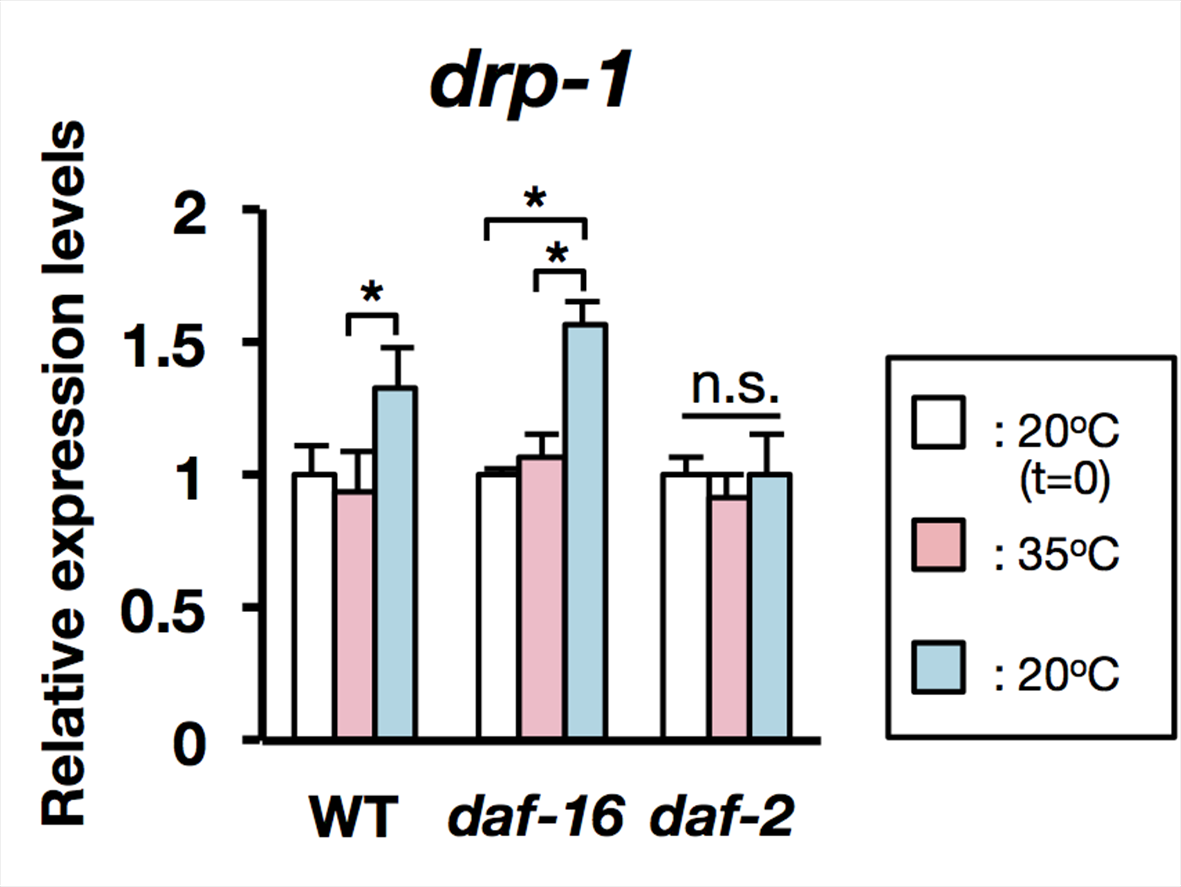

Supplement: Supplementary file 3 [file 1985FigureS2.tiff]
